# All-Optical Switching Demonstrated with Photoactive Yellow Protein Films

**DOI:** 10.3390/bios11110432

**Published:** 2021-10-31

**Authors:** Dániel Petrovszki, Szilvia Krekic, Sándor Valkai, Zsuzsanna Heiner, András Dér

**Affiliations:** 1 Institute of Biophysics, Biological Research Centre, Eötvös Loránd Research Network, 6726 Szeged, Hungary; petrovszki.daniel@brc.hu (D.P.); krekic.szilvia@brc.hu (S.K.); valkai.sandor@brc.hu (S.V.); 2 Doctoral School of Multidisciplinary Medical Sciences, University of Szeged, 6720 Szeged, Hungary; 3 School of Analytical Sciences Adlershof, Humboldt-Universität zu Berlin, 12489 Berlin, Germany; heinerzs@hu-berlin.de

**Keywords:** optical switching, integrated optics, photonics, photoactive yellow protein

## Abstract

Integrated optics (IO) is a field of photonics which focuses on manufacturing circuits similar to those in integrated electronics, but that work on an optical basis to establish means of faster data transfer and processing. Currently, the biggest task in IO is finding or manufacturing materials with the proper nonlinear optical characteristics to implement as active components in IO circuits. Using biological materials in IO has recently been proposed, the first material to be investigated for this purpose being the protein bacteriorhodopsin; however, since then, other proteins have also been considered, such as the photoactive yellow protein (PYP). In our current work, we directly demonstrate the all-optical switching capabilities of PYP films combined with an IO Mach–Zehnder interferometer (MZI) for the first time. By exploiting photoreactions in the reaction cycle of PYP, we also show how a combination of exciting light beams can introduce an extra degree of freedom to control the operation of the device. Based on our results, we discuss how the special advantages of PYP can be utilized in future IO applications.

## 1. Introduction

Integrated optics (IO) is a new alternative method of information transfer analogous to integrated electronics; however, the speed of the system at hand is dependent on the nonlinear optical (NLO) material that is applied as the active element of the IO circuit. Several materials are being developed and used in hybrid systems—mostly nonlinear crystals with π-conjugated electron systems [1,2]. It is among the long-term goals in optical telecommunication to find proper NLO materials that make possible all-optical IO switching at the proper efficiencies and speeds. Earlier works suggested the consideration of materials of biological origin for these purposes [3]. First, it was shown that (slow) spectral changes accompanying the photocycle of the chromoprotein bacteriorhodopsin (bR) are sufficient to achieve IO switching [4,5,6], making bR a promising candidate for IO applications. Eventually, it was also demonstrated that the primary events of the bR photocycle allow for ultrafast (sub-picosecond) switching as well [7]. 

The application of biological materials is appealing because of their easy availability and exceptional NLO properties [8]. Recently, another light-sensitive biomaterial, the photoactive yellow protein (PYP) [9,10], has garnered interest for IO applications because of its fast photocycle in solution and its large light-induced refractive index change in dried films [11]. PYP, being water-soluble and smaller than the bR membrane patches used previously, potentially enables its combination with special IO passive elements where the application of bR is not possible (e.g., in porous silicon structures). Based on our previous experiments, the PYP in film form could be a viable option for all-optical switching experiments, as adding glycerol to the protein solution before drying the film helps maintain the integrity of the photocycle even in low humidity environments, allowing for the formation of the intermediate states [12], which accompanies refractive index changes of the film [11]. The photocycle of PYP consists of two main intermediates, the red-shifted pR and the blue-shifted pB (further distinguishing pR_1_, pR_2_, pB_1_ and pB_2_) [10]. By illuminating PYP with blue light, the photocycle takes place in a matter of milliseconds in solution. A study has shown that illuminating PYP during the photocycle’s pB intermediate with violet light can cause a short circuit in the photocycle, making PYP return to the ground state via a faster route [13]. 

In this communication, our main motivation was to demonstrate that the spectral changes of the PYP photocycle can accompany refractive index changes sufficient for IO switching (similar to how it has been shown earlier for bR [4]), using the combination of a PYP film as an active NLO component and a proper integrated optical structure. Active IO elements can be implemented by utilizing a number of structures—interferometers [8,14], grating couplers [14,15], ring resonators [16], etc. The Mach–Zehnder interferometer (MZI) is one of the simplest IO passive devices, consisting of a bifurcated linear IO waveguide structure forming two arms. By adsorbing a transparent NLO material on top of the arms, adlayers are formed that can alter the effective refractive index, thus creating a phase difference between the arms joining at the output of the device, manifesting in intensity changes at the MZI’s output. This principle is often used for biosensing applications, such as sensing bacteria or proteins in action, as well [17,18]. Hence, we performed all-optical switching experiments using an IO MZI, with a PYP adlayer as an active NLO component, for the first time. For the excitation of the sample, we used continuous illumination with two different laser wavelengths, targeting both the protein’s pG ground state and pB intermediate state, demonstrating the different modalities of the switching capability of PYP-containing IO devices. Our results underpin that, besides bR, the chromoprotein PYP can also be considered as a promising NLO material for future, high-profile IO applications.

## 2. Materials and Methods

### 2.1. PYP Sample Preparation

The preparation method for PYP has been discussed elsewhere [10,11], but a brief summary will be given here as well. To prevent the cracking of the PYP films, 87% glycerol solution was added to the protein solution at 1:49 ratio. It was then allowed to dry in a laboratory environment (33% RH, 20 °C) for at least 24 h. By using glycerol as a ballast material, we secured the relative humidity inside the sample at ~80%, still allowing for the photocycle to take place [10,11].

### 2.2. IO Mach–Zehnder Interferometer Biosensor Fabrication

The fabrication method of the integrated optical Mach–Zehnder interferometer was based on the process used in one of our previous works [19], following the protocol of the manufacturer of the applied materials. The mentioned process was modified, considering the parameters used in several steps, to reach the desired thickness of the components of the device. 

**Figure 1:**
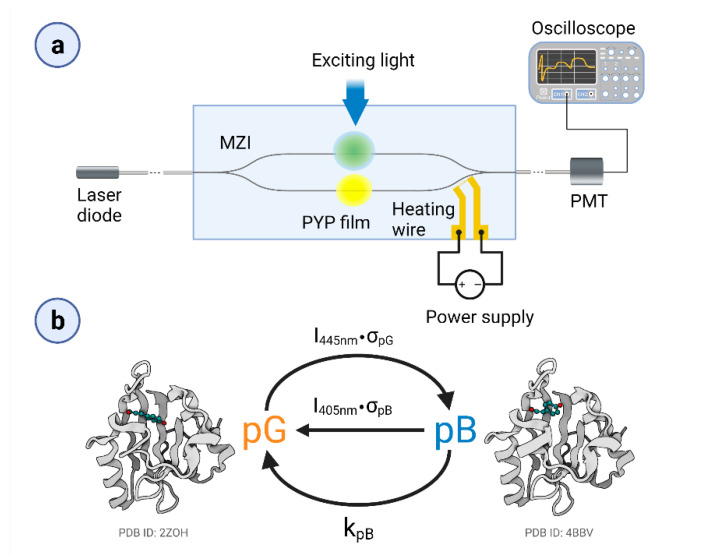
(**a**) Schematic representation of the measuring IO device, the MZI with the PYP adlayer and the heating wire for bias-point adjustment. (**b**) Simplified photocycle of the PYP film, with the ground state (pG) and the rate-limiting intermediate (pB). (Schematic PDB-structures are indicated for demonstration.) Long, continuous excitation with blue light results in the development of a pG–pB equilibrium. The figure was created by Biorender.com.

It should be kept in mind that MZIs have a sinusoidal transmission function (TMF), allowing for an approximate linear response of the device only in the vicinity of the inflexion points of the TMF and that this is where the bias point should be adjusted prior to the measurements. There are several methods to accomplish this task, in most cases via tuning the optical path length along one of the arms. Previously, we have used an optical solution [19]; however, here, controlled heating near one of the branches of the MZI was applied. By using a surface-sputtered heating wire close to the reference arm of the MZI, we can modify the environment’s temperature and thus tune the device’s bias point. According to our experience, the heating effects remained restricted to the area of the heating wire that was more than 1600 µm away from the proximal adlayer area (and even further away from the distal ones), leaving enough space for dissipation. This solution resulted in an improved stability of the bias point during the measurements. In order to accomplish this task, a glass substrate covered by a gold heating wire structure was used, based on the method applied for surface electrode fabrication [19]. As a first step of the device fabrication, a 20 nm thick surface gold heating wire (1 kΩ resistance) capable of performing MZI’s bias-point tuning was prepared on a microscope coverslip (Menzel-Gläser, Thermo Fisher Scientific, Waltham, MA, USA). Then, a rib waveguide stripe (SU-8 2002, MicroResist Technology GmbH., Berlin, Germany) of 2 × 2 μm was made on the mentioned glass substrate, forming the sensing optical interferometer structure in such a way that one of the arms was placed in the vicinity of the wire, as can be seen in Figure 1a.

In the device’s ready-to-use construction, the substrate was glued on a microscope slide (Menzel-Gläser, Thermo Fisher Scientific, Waltham, MA, USA) using NOA81 optical adhesive by exposing the layer with a mercury arc lamp (100W, HBO 100 Zeiss, Jena, Germany). To apply the tuning capability of the heating wire, electric wires were connected by droplets of conducting epoxy (CW2400 CircuitWorks^®^ Conductive Epoxy, Chemtronics, Kennesaw, GA, USA) to the side contact pads of the gold structure. These were connected to the DC power supply (VLP 2403pro, Conrad Electronic, Hirschau, Germany), to perform the bias-point tuning.

### 2.3. Experimental Setup

A schematic representation of the experimental setup can be seen in Figure 1a. As a measuring light (referred to as a probe light), we used a green laser diode (532 nm, 50 mW, Roithner, Wien, Austria), which was coupled inside the single mode MZI by a single-mode optical fiber (S630-HP, Thorlabs GmbH, Lübeck, Germany). The fiber was positioned to the MZI’s input with a micropositioner (DC-3K, Märzhäuser Wetzlar GmbH & Co. KG, Steindorf, Germany) and its optimal position was fixed with photopolymer glue (OP-66-LS, Dymax Europe GmbH, Wiesbaden, Germany). The same method was used for coupling the light out from the device. The stock solution of the PYP–glycerol mixture was pipetted on both arms of the interferometer, in ~1 mm diameter patches, before drying. To excite and control the PYP photocycle, we used two different continuous laser beams (445 nm, 4/44 mW and 405 nm, 21.7 mW at the sample). The scheme of the simplified photocycle model is shown in Figure 1b. The duration of excitation was varied between 2 and 14 s. PYP films were deposited on both arms of the MZI but only one of them was excited at a time. For tuning the MZI’s bias point, the voltage of the heating wire was varied between 0–4.6 V.

To measure the transfer characteristics of the MZI, its output was coupled to an optical fiber guiding the measuring light into a photomultiplier tube (H5783-01, Hamamatsu, Japan), from which the signal was transmitted to and recorded by a digital oscilloscope (LeCroy 9310-L, LeCroy, Chestnut Ridge, NY, USA). The voltage on the heating wire was controlled by a variable DC power supply (VLP 2403pro, Konrad Electronics, Hirschau, Germany). During each measurement, the ambient temperature was kept at 23 °C, with a relative humidity of 33%.

## 3. Results

### 3.1. Calibration of the MZI Bias Point 

**Figure 2:**
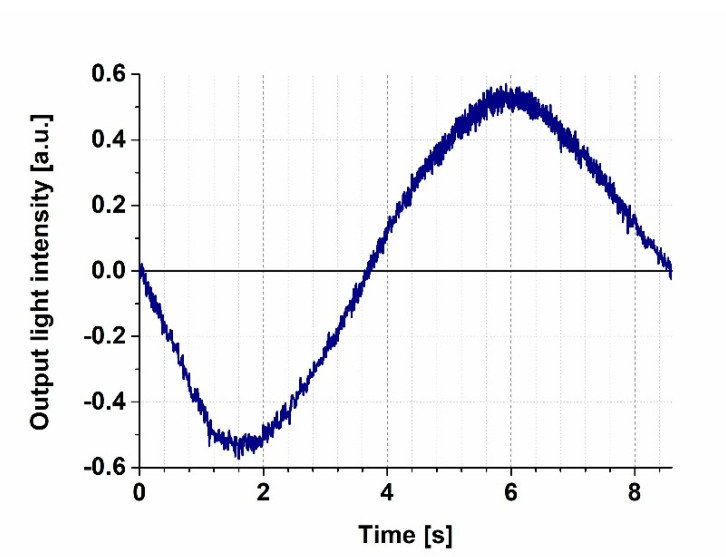
Transmission function of the MZI recorded by a 532 nm probe light, while increasing the heating voltage steadily from 0 to 4.6 V. Prior to each switching experiment, the bias point was adjusted to the zero level.

We first measured the transmission characteristics of our MZI by steadily increasing the voltage applied to the heating wire from 0 V to 3.5 V while detecting the intensity of the 633 nm probe light at the output of the interferometer (Figure 2). The heating caused a local thermal dilatation of the nearby waveguide area, resulting in an increase of the effective optical path length of the reference arm of the MZI, thereby introducing a phase difference between the light beams interfering with each other at the joint of the output side. The MZI’s sensitivity is proportional to the first derivative of the sinusoidal transmission function, meaning that the sensitivity is smaller at the extremes, while it is the highest at the mean light intensity, compensated to an artificial zero output level by an offset voltage of the amplifier (“zero-intensity points” in Figure 2), where the derivative is the largest. By fine-tuning the power dissipated by the heating wire, one can adjust the bias point of the interferometer, before measurements, to one of these points of maximal sensitivity. One should note that changes in the ambient conditions (such as temperature and humidity) might cause a baseline drift (and an accompanying sensitivity change), so a careful control of these parameters is necessary to perform the experiments. In our case, the baseline drift could be kept on a negligible level during the time scale of the experiments (typically the 10-s to several minutes scale) by using the method of stationary local electric heating. The slight baseline drifts that still occurred during the present experiments could be attributed, rather, to local heating effects due to the several-second-long illumination of PYP films. Note that similar phenomena were observed while performing analogous experiments with bR, too [4]. Such effects, however, should play even less of role in future fast-switching experiments.

### 3.2. Demonstration of All-Optical Switching

**Figure 3:**
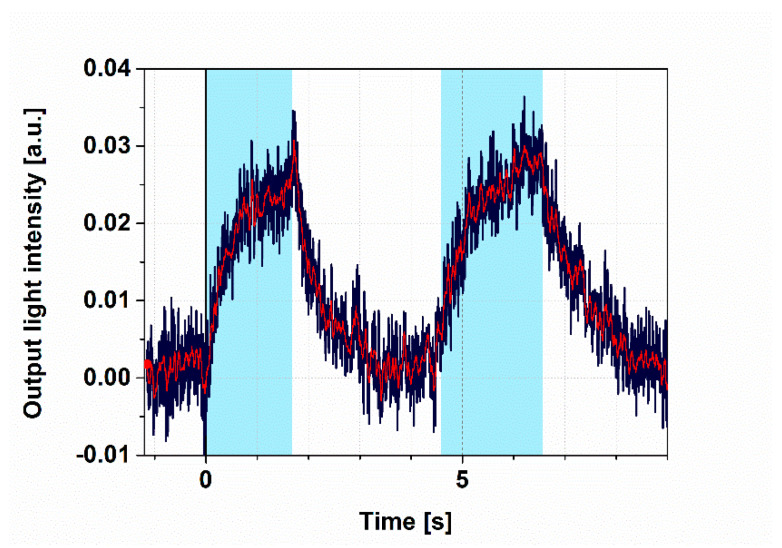
Demonstration of switching I. Light intensity measured at the MZI’s output with two consecutive 4 s square-wave excitations. Excitation light: 445 nm, 4 mW; probe light: 532 nm; duty cycle: 50%; heating voltage: 4 V for adjusting the bias point. The blue line represents the original signal, while the red line corresponds to the filtered one. Cyan color shades indicate the periods of 445 nm illumination.

To demonstrate all-optical switching, the intensity changes of the probe light were monitored, while PYP was excited by 405 nm continuous illumination. Prior to illumination, the bias point was properly adjusted to the “zero” level (Figure 2) by tuning the heating voltage to 4 V and keeping it constant during the whole period of detecting the output intensity of the MZI over time. The wavelength of the probe light was chosen to be 532 nm, so as to stay outside of the absorption range of the ground state and the intermediates of the PYP photocycle, respectively (Figure S2a), while still being in the high-refractive-index regime near the absorption peaks (Figure S2c). We used a 2 s long illumination and a subsequent 2 s break, during which no exciting light reached the sample. The significant changes of the measuring light level between the light and dark periods indicate that all-optical switching works properly (Figure 3) and that the bias point at 4 V heating voltage suffered only negligible drift. According to a brief interpretation of the observed phenomena based on a simplified reaction scheme of the PYP photocycle (Figure 1b), a steady-state equilibrium between the ground state (pG) and the rate-limiting intermediate (pB) was formed about 2 s after excitation, in accordance with our earlier results [11], establishing a corresponding refractive index change of the protein-film adlayer (Figure S2c). After another 2 s of the dark period, most of the protein returned to the ground state, which, upon re-excitation, yielded the same signal as when exciting the sample for the first time (Figure 3), demonstrating the repeatability of switching. (A more detailed explanation of the effects is given below, in the Discussion section.)

### 3.3. Controlling the Photocycle of PYP by Two Excitation Lights

The photocycle of PYP contains at least one light-induced shortcutting route, namely, a preferential excitation of pB drives back the molecule to the pG state [13], as shown schematically in Figure 1b. This phenomenon offers another degree of freedom to control PYP-based all-optical switching. Here, we demonstrate this opportunity by a combination of quasi-permanent illuminations. 

**Figure 4:**
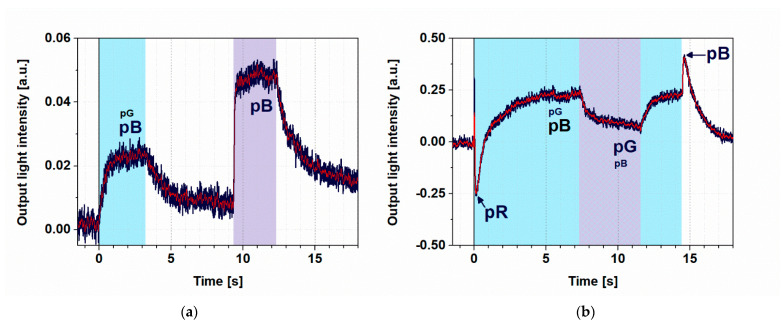
Demonstration of switching II. (**a**) Light intensity measured at output of the MZI, when the sample was first illuminated by a 445 nm (4 mW) excitation beam for 3 s. Then, after a dark period of 6 s, PYP was re-excited with a 405 nm illumination (27 mW) for 3 s. The bias voltage was set to 2.1 V. (**b**) Measured output signal of the MZI with 14 s excitation by a strong (44 mW) and continuous 445 nm light while, from 7 s, also exciting the film for 4 s by a 405 nm continuous light. The blue line represents the measured data, while the red is the filtered curve. The wavelength of the probe light propagating in the MZI was 532 nm in both cases. Color shades indicate the periods and wavelengths of illumination (cyan and violet for 445 nm and 405 nm, respectively, striped pattern for both). To facilitate understanding of the observed effects, we have now also indicated the states of the photocycle of PYP contributing to a greater or lesser extent (indicated by the size of the caption) to the actual effect, represented by different phases of the registered traces.

First, we excited the PYP film with a weak (4 mW), 445 nm light for 3 s, which was followed by a relaxation in the dark, then another 3 s excitation at 405 nm (Figure 4a). Here, we can see that illuminations at both wavelengths were able to effectively excite the protein in the ground state and accumulate pB, as explained before, in agreement with its absorption spectrum (Figure S1a). The size difference of the two signals can be attributed to the much higher intensity of the 405 nm light (27 mW) that was overcompensating for the higher absorption cross section at 445 nm (4 mW). 

When, however, a strong 445 nm excitation was used (44 mW), a higher-level steady state was formed and the subsequently superimposed 405 nm excitation resulted in a quenching of the effect (Figure 4b), demonstrating that switching effects based on PYP films can be further controlled by a combination of illuminations. A more detailed explanation of the kinetic effects is given in the next section. 

## 4. Discussion

### 4.1. Model Calculations

To interpret the registered kinetic traces, let us consider a typical scheme for photoexcitable disordered systems, where a primary absorption is followed by thermally driven processes, such as the stabilization of the light-induced conformational change and a subsequent relaxation [20,21,22]. While a single-photon absorption process is considered extremely fast, on the level of population of atoms or molecules the primary reaction is described by a rate constant that is proportional to the excitation light intensity (I) and an efficiency factor (σ), including the absorption cross section; on the other hand, the thermal processes are assumed to be light-independent [7,20,21,22]. This general scheme holds for light-driven chromoproteins as well; however, here, the thermally induced relaxation process is usually more complex than, e.g., in semiconductors [7]. (For PYP, such a complex, though not complete, model is shown in Figure S1 and the corresponding kinetic equations are shown in Supporting Information.) Note, however, that in a first-order unidirectional reaction scheme, the slowest (rate-limiting) transitions dominate the kinetics. If, e.g., the I·σ for a light-driven reaction is much smaller than the rate constants of the subsequent thermal transitions, the contribution of the latter to the transient or equilibrium population of the intermediate states is negligible. Hence, at the relatively low light intensity levels provided by the continuous-wave lasers used in our experiments, we consider a simplified photocycle scheme, keeping only the ground state (pG) and the rate-limiting pB intermediate, accumulating to the largest extent under such circumstances (Figure 1b). (The differential equation system describing the kinetics is also reduced in this case, accordingly.) For the light-induced reactions under stationary illumination at 445 and 405 nm, we can introduce the quasi-first-order rate constants I_445nm_·σ_pG_ and I_405nm_·σ_pB_, respectively. (Here I_445nm_ and I_405nm_ denote the continuous blue and violet light intensities, while σ_pG_ and σ_pB_ include the absorption cross sections and the quantum efficiencies of the light-induced photocycle reactions at 445 and 405 nm, respectively.) The rate constant of the thermally induced decay of the pB intermediate is denoted by k_pB_. Under continuous blue-light illumination (and in the absence of violet light), the equilibrium concentration of pG and pB can be expressed as follows:

(1) $$\left[\mathrm{pB}\right]={\mathrm{PG}}_{0}\cdot I_{445\mathrm{nm}}\cdot \frac{\sigma_{\mathrm{pG}}}{I_{445\mathrm{nm}}\cdot \sigma_{\mathrm{pG}}+{\;k}_{\mathrm{pB}}}\;\mathrm{and}\;\left[\mathrm{pG}\right]={\;\mathrm{PG}}_{0}\cdot I_{445\mathrm{nm}}\cdot \frac{k_{\mathrm{pB}}}{\left({\;I}_{445\mathrm{nm}}\cdot \sigma_{\mathrm{pG}}+{\;k}_{\mathrm{pB}}\right)},$$

where [pG] and [pB] are concentrations of the ground state and the pB intermediate, respectively, while PG_0_ = [pG] + [pB] and d[pG]/dt = d[pB]/dt = 0.

If both the blue and the violet lights are present, the photocycle scheme becomes somewhat more complex: the pG to pB transition will now be driven by the sum of two rate constants (I_445nm_·σ_pG_ + I_405nm_·σ_pG405_), where σ_pG405_ stands for the absorption cross section of pG at 405 nm. The apparent rate constant of the pB to pG transition will be similarly modified to I_445nm_·σ_pB445nm_ + I_405nm_·σ_pG_. It is, however, evident from Figure S2a, that σ_pG405_ < σ_pG_, while on the other hand, σ_pB445nm_ << σ_pB_. Being the blue and violet excitation light intensities of the same order of magnitude, we can end up with the following formula for the approximate concentrations of PYP states under two-color excitation:

(2) $$\left[\mathrm{pB}\right]={\;\mathrm{PG}}_{0}\cdot I_{445\mathrm{nm}}\cdot \frac{\sigma_{\mathrm{pG}}}{\left(I_{445\mathrm{nm}}\cdot {*\sigma }_{\mathrm{pG}}+{\;I}_{405\mathrm{nm}}\cdot \sigma_{\mathrm{pB}}+{\;k}_{\mathrm{pB}}\right)},\;\mathrm{and}\;\left[\mathrm{pG}\right]={\;\mathrm{PG}}_{0}\cdot I_{445\mathrm{nm}}\cdot \frac{k_{\mathrm{pB}}}{\left(I_{445\mathrm{nm}}\cdot \sigma_{\mathrm{pG}}+{\;I}_{405\mathrm{nm}}\cdot \sigma_{\mathrm{pB}}+{\;k}_{\mathrm{pB}}\right)}.$$

When exciting the pG ground state—which has an absorption maximum at 446 nm—with sufficiently high light intensity (i.e., if I_445nm_·σ_pG_ >> k_pB_), a steady-state equilibrium between pG and pB is established according to (1), with the PYP primarily being in the pB state, since the $I_{445\mathrm{nm}}\cdot \sigma_{\mathrm{pG}}$ contribution is dominating the k_pB_ kinetic constant. From what we have seen in previous experiments, too [12], the steady state forms in the matter of a few seconds under such conditions, while the protein returns fully to the ground state after excitation ceases. If we use two different wavelengths for exciting the sample, choosing one to target, rather, pG, and another to target, rather, the pB intermediate, we can shift the concentration ratio of intermediates in the formed steady state. By introducing $I_{405\mathrm{nm}}\cdot \sigma_{\mathrm{pB}}$ into the rate equation we preferentially excite the dominant pB state, inducing a shortcut reaction back into pG [13]. The equilibrium concentration of pB and pG in this case are given by equation (2). The absorption spectra of the ground and intermediate states of the photocycle are shown in Figure S2a, from which, the corresponding difference absorption spectra can be determined (Figure S2b) and the refractive index difference spectra can be calculated according to the Kramers–Kronig relations [23] (Figure S2c). 

The corresponding light-induced refractive index change of the PYP adlayer adsorbed on the MZI induces a phase shift in the measuring arm of the MZI, resulting in an intensity change at the output. The steady state, formed by using both excitation wavelengths simultaneously, has a different refractive index than that formed by illuminating the film at only one wavelength. This phenomenon makes a detectable output intensity difference between the two states, which is utilized in our switching experiments. The transient positive and negative peaks in Figure 4b must have different routes. The latter we attribute to a transient accumulation of pR, a red-shifted photocycle intermediate (Figure S1), at high 445 nm intensities before decaying to pB, dominating the steady state [10]. On the other hand, the positive peak followed by the switch-off is probably due to a shortcutting effect of the 445 nm light itself, exciting the accumulating intermediate(s) at high intensities. 

### 4.2. Evaluation of Kinetics

Although, in the equations presented in this chapter, we deal only with steady-state concentrations and did not intend to solve the complex, coupled differential equation system describing the photocycle kinetics (Supporting Information), some straightforward statements can still be made for the kinetics of rate-limiting reactions. In case of all transitions to the dark state after illumination, e.g., the slowest rate of the thermal reactions of the photocycle, namely, the pB to pG transition, is supposed to determine the time constant of relaxation. In fact, the results of the exponential fitting of these phases in Figure 3 and Figure 4 all show a similar value within the estimated uncertainty limits of the experiments and evaluation (1.2 ± 0.2 s). This value is actually also congruent with the findings of earlier measurements on similar samples [12]. In the case of transitions to light-driven equilibria of various photocycle intermediates, on the other hand, the observed rates are always a mixture of virtual rate constants, due to the illumination driving the initial population towards a new equilibrium (I*σ) and a thermal rate constant (k), i.e., k_virtual_ = I*σ + k, as is discussed above, too. This means that these transitions should normally be faster than the ones discussed before. In fact, fitting the rising phases of the light-induced signals yields shorter time constants (τ_rise_) than the ones corresponding to relaxations. In addition, the rates belonging to the same transitions normally increase by increasing intensity of excitation, as expected from the above equation (τ_rise_ = 600 ± 100 ms for the 4 mW excitation at 445 nm and τ_rise_ = 200 ± 40 ms for the 27 mW excitation at 405 nm. (Note that in the latter case, the higher intensity overcompensated the smaller absorption cross section (σ).) The kinetics of the PYP photocycle, however, get more complex when light-induced back-reactions are also involved, either due to a simultaneous excitation at two wavelengths, or to an excitation of more than one intermediate by the same excitation light (Figure 4b). The latter occurs, e.g., when a high-intensity illumination can compete with the pR to pB decay, resulting in the accumulation of pR. However, because of the highly overlapping spectra of pG and PR, the 445 nm excitation light drives back a considerable population of pR to pG before being able to get further to pB, following the normal pathway of the photocycle. This effect actually results in a slow-down of the formation of pB populations, even at high intensities of the excitation light. Right after switching off the strong 445 nm illumination (Figure 4b, near 14 ms), however, we see a fast rise (40 ms), which is due to the population of molecules that have been driven out from the ground state (pG) and, in the absence of a back-reaction, reach the pB state rapidly.

Although establishing a comprehensive model for the in-depth, quantitative description of the phenomena is beyond the scope of this short, application-oriented study, we could show that the PYP photocycle can be controlled by various illuminations. This allows for a sort of dynamic “programing” of PYP-based NLO materials by light, a potentially utilizable feature in future IO applications. According to our experiments, the light-induced intensity changes accompanying the PYP photocycle cover a major part of the full dynamic range of the Mach–Zehnder interferometer (Figure 2 and Figure 4b), clearly proving the IO switching ability of PYP-based NLO films. Comparing the results with those of switching experiments carried out on a Mach–Zehnder interferometer structure doped by bR films also shows that the quality of the traces obtained by the two experiments are rather similar [4].

### 4.3. On the Opportunity of Increasing Switching Speed

Based on the present results and those of some recent publications [12,24], it can also be envisioned that, similarly to the case of bR, ultrafast all-optical switching by PYP films as NLO materials should be feasible. On the one hand, the kinetics analysis of our results under 4.2 implies that increasing the intensity of the excitation light increases the switching speed as well. However, we also saw that at higher light intensities, one should consider a more detailed photocycle scheme, including the faster-forming and decaying intermediates (Figure S1). On the other hand, Konold et al. have recently shown that large absorption changes occur in PYP samples after a strong, 50 fs excitation, still in the femtosecond time scale [24], where a blue-shifted intermediate (called ”ES”, after ”excited state”) develops. On the ns to µs time scale, it transforms to a red-shifted intermediate (pR) (Figure S1), which is known to transform later to a blue-shifted pB in the course of milliseconds [25] at room temperature (Figure S1). The absorption spectra of the pR and pB intermediates have been determined from kinetic experiments [25] (Figure S2a). From the Kramers–Kronig relations of optics, connecting the real and imaginary parts of the complex refractive index, one can then calculate the corresponding refractive index spectra [23] (Figure S2c). Although the full absorption spectrum of the ES intermediate has not been published yet, the relative sizes of the light-induced differential spectra of ES, pR and pB (Figure S2b) imply that the amplitude of the refractive index change associated to the pG to ES transition will most probably exceed those of the pG to pR and pG to pB transitions. Since the results of the present paper prove that refractive index changes associated with the pG to pB transition are sufficient for integrated optical switching, it can be safely stated that it should be also true for the pG to ES transition, which takes place on the femtosecond time scale, similar to the bR-I transition of bacteriorhodopsin [7]. Hence, it can be anticipated that an ultrafast integrated optical switching based on the primary phototransition of PYP should also be feasible to demonstrate at high-intensity short-pulse excitations with a proper femtosecond setup.

## 5. Conclusions and Outlook

Our results demonstrate that dried films of PYP can be used for all-optical IO switching because of the favorable nonlinear optical properties of the protein film. For PYP, being water-soluble and smaller than the bR membrane patches used previously theoretically enables its combination with special IO passive elements where the application of bR is not possible (e.g., with porous silicon—pSi—structures). According to a solid functionalization protocol developed for pSi structures, they can accommodate a variety of soluble proteins; as it has already been demonstrated for a couple of proteins of various sizes and compositions [26,27], so it is expected to be achievable for PYP as well. 

The small size and water solubility are not the only properties that distinguish PYP from bR and allow for its unique applications. Another important feature is the different spectral range of the main absorption bands and refractive index changes, which are blue shifted in PYP by ca. 100 nm as compared to bR, making a complementary spectral range available for IO operations using biophotonic film as active, nonlinear optical materials.

For utilization in telecommunication, however, much faster switching procedures are usually required, since the present state of the art for solid-state NLO materials is in the subnanosecond range [28]. Similarly to the case of bacteriorhodopsin [7], the light-induced primary photocycle reactions of PYP [24] theoretically allow for such a short switching time regime, whose demonstration should be the subject of follow-up papers.

## Data Availability

The original data are available upon request from the corresponding author.

## Supplementary Materials

The following are available online at https://www.mdpi.com/article/10.3390/bios11110432/s1, Figure S1. A simplified scheme of the PYP photocycle, including the light-induced and thermal reactions, represented by undulated and solid arrow lines with the corresponding rate constants, respectively. I and σ represent the light intensity and the absorption cross sections, pG stands for the ground-state pigment and ES is the blue-shifted first excited state forming on the 10 fs time scale at high I intensities and decaying to the red-shifted pR on the nanosecond to microsecond time scale (Konold et al.). pR decays further to the blue-shifted pB on the 1 ms to 100 ms time scale (Khoroshyy et al., Krekic et al., 2019). Both pR and pB can be driven back by photoexcitation to pG. A differential equation system describing the kinetics of the concentrations of the photocycle intermediates is also presented. Figure S2. (a) Absorption spectra of the ground state (pG, black), an early intermediate (pR, red) and the rate-limiting intermediate (pB, blue) of the PYP photocycle, as determined from absorption kinetic experiments (Khoroshyy et al.). (b) Difference-absorption spectra of pR (red) and pB (blue), as compared to the ground state (pG). (c) Calculated change of the refractive index spectrum upon the rate-limiting pG → pB transition (underlying the effects described in the main body of the paper), using the Kramers–Kronig relations. For more details, see Fábián et al.
