# Supplementary material for: All-Optical Switching Demonstrated with Photoactive Yellow Protein Films
Source: Biosensors (Basel). 2021 Oct 31;11(11):432. doi: 10.3390/bios11110432 (PMC8615624; doi:10.3390/bios11110432)
Supplement: Supplementary file 1 [file biosensors-11-00432-s001.zip › biosensors-1385218-supplementary.pdf]

Supplementary Information

All-Optical Switching Demonstrated with Photoactive Yellow Protein Films

Dániel Petrovszki [1,2,†], Szilvia Krekic [1,2,†], Sándor Valkai [1], Zsuzsanna Heiner [3] and András Dér [1,*]

[1] Institute of Biophysics, Biological Research Centre, Eötvös Loránd Research Network, 6726 Szeged, Hungary; petrovszki.daniel@brc.hu (D.P.); krekic.szilvia@brc.hu (S.K.); valkai.sandor@brc.hu (S.V.)

[2] Doctoral School of Multidisciplinary Medical Sciences, University of Szeged, 6720 Szeged, Hungary

[3] School of Analytical Sciences Adlershof, Humboldt-Universität zu Berlin, 12489 Berlin, Germany; heinerzs@hu-berlin.de

* Correspondence: der.andras@brc.hu

† These authors contributed equally to this work: Dániel Petrovszki and Szilvia Krekic.

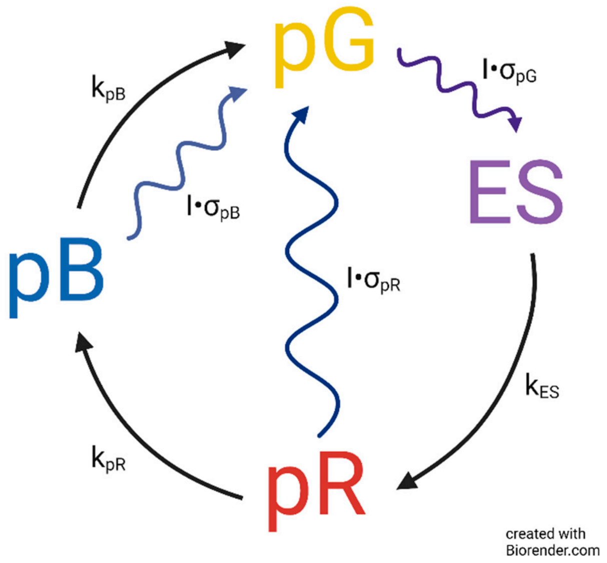

Figure S1. A simplified scheme of the PYP photocycle, including the light-induced and thermal reactions, represented by undulated and solid arrow lines with the corresponding rate constants, respectively. I and σ represent the light intensity and the absorption cross sections, pG stands for the ground-state pigment, ES is the blue-shifted first excited state forming on the 10-fs time scale at high I intensities, and decaying to the red-shifted pR on the nanosecond to microsecond time scale [Konold et al.]. pR decays further to the blue-shifted pB on the ms to 100-ms time scale [Khorosyy et al., Krekic et al., 2019]. Both pR and pB can be driven back by photoexcitation to pG. .

The differential equation system describing the kinetics of the concentrations of the photocycle intermediates:

$$\frac{d[pG]}{dt} = -I \cdot \sigma_{pG} \cdot [pG] + I \cdot \sigma_{pR} \cdot [pR] + I \cdot \sigma_{pG} \cdot [pG] + \left(I \cdot \sigma_{pB} + k_{pB}\right) \cdot [pB]$$

$$\frac{d[ES]}{dt} = I \cdot \sigma_{pG} \cdot [pG] - k_{ES} \cdot [ES]$$

$$\frac{d[pR]}{dt} = k_{ES} \cdot [ES] - \left(I \cdot \sigma_{pR} + k_{pR}\right) \cdot [pR]$$

$$\frac{d[pB]}{dt} = k_{pR} \cdot [pR] - \left(I \cdot \sigma_{pB} + k_{pB}\right) \cdot [pB]$$

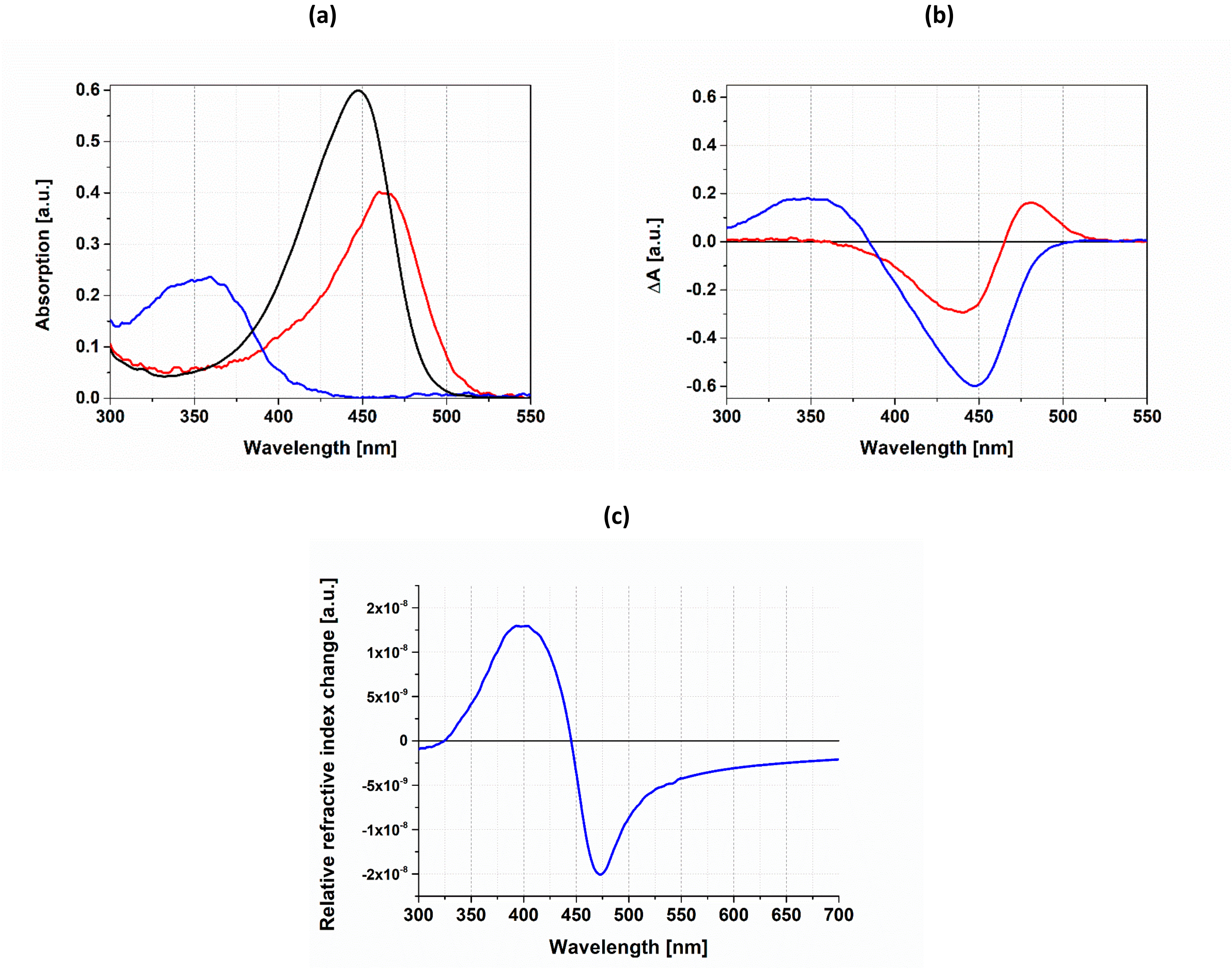

Figure S2. a) Absorption spectra of the ground state (pG, black), an early intermediate (pR, red) and the rate-limiting intermediate (pB, blue)) of the PYP photocycle, as determined from absorption kinetic experiments [Khoroshyy et al.]. **b)** Difference-absorption spectra of pR (red) and pB (blue), as compared to the ground state (pG). **c)** Calculated change of the refractive index spectrum upon the rate-limiting pG → pB transition (underlying the effects described in the main body of the paper), using the Kramers-Kronig relations. For more details, see [Fábián et al.].

References

1. Konold, P.E., et al., Confinement in crystal lattice alters entire photocycle pathway of the Photoactive Yellow Protein. Nature Communications, 2020. **11**: p. 4248.
2. Khoroshyy P. et al., Effect of Hofmeister cosolutes on the photocycle of photoactive yellow protein at moderately alkaline pH. J. Photochem. Photobiol. B, 2013, **120**: 111
3. Krekic, S., et al., Spectrokinetic characterization of photoactive yellow protein films for integrated optical applications. European Biophysics Journal, 2019, **48**: 465–473
4. Fábián, L., et al., *Integrated optical investigation of two light-sensitive proteins.* AIP Conference Proceedings, 2017. **1796**(1): p. 040001.
